# EoML-SlideNet: A Lightweight Framework for Landslide Displacement Forecasting with Multi-Source Monitoring Data

**DOI:** 10.3390/s25175376

**Published:** 2025-09-01

**Authors:** Fan Zhang, Yuanfa Ji, Xiaoming Liu, Siyuan Liu, Shuai Ren, Xizi Jia, Xiyan Sun

**Affiliations:** 1 School of Information and Communication, Guilin University of Electronic Technology, Guilin 541004, China; 21021101008@mails.guet.edu.cn (F.Z.); jiyuanfa@guet.edu.cn (Y.J.); 18907830034@163.com (X.J.); 2 Department of Internet of Things Engineering, School of Computer Science and Engineering, Guilin University of Aerospace Technology, Guilin 541004, China; 3 Guangxi Zhuang Autonomous Region Geological Environment Monitoring Station, Nanning 530201, China; 15277405178@163.com; 4 School of Computer Science and Information Security, Guilin University of Electronic Technology, Guilin 541004, China; 21031102009@mails.guet.edu.cn; 5 Faculty of Electrical and Electronics Engineering Technology, Universiti Malaysia Pahang Al-Sultan Abdullah, Pekan 26600, Malaysia; rs0066good@gmail.com

**Keywords:** landslide displacement forecasting, EoML-SlideNet, DBLE-LV, FLOPs, inference time, lightweight edge-level modeling

## Abstract

The karst terrain of Guangxi, China, characterized by steep slopes and thin residual soils, is highly vulnerable to rainfall-induced shallow landslides. Timely and accurate displacement forecasting is critical for early warning and risk mitigation. However, most existing systems depend on centralized computation, leading to latency and reduced responsiveness. Moreover, conventional forecasting models are often too computationally intensive for edge devices with limited processing resources. To address these constraints, we present EoML-SlideNet, a lightweight forecasting framework designed for resource-limited hardware. It decomposes displacement and triggers into trend and periodic components, then applies the Dual-Band Lasso-Enhanced Latent Variable (DBLE–LV) module to select compact, interpretable features via cross-correlation, LASSO, and VIF screening. A small autoregressive model predicts the trend, while a lightweight neural network captures periodic fluctuations. Their outputs are combined to estimate displacement. All models were evaluated on a single CPU-only workstation to ensure fair comparison. This study introduces floating-point operations (FLOPs), alongside runtime, as practical evaluation metrics for landslide displacement prediction models. A site-specific multi-sensor dataset was developed to monitor rainfall-triggered landslide behavior in the karst terrain of Guangxi. The experimental results show that EoML-SlideNet achieves 2–4 times lower MAE/RMSE than the most accurate deep learning and the lightest baseline models, while offering 3–30 times faster inference. These results demonstrate that low-complexity models can match or surpass the accuracy of deep networks while achieving latency and FLOP levels suitable for edge deployment without dependence on remote servers.

## 1. Introduction

Landslides are a persistent threat to life and infrastructure, especially in areas prone to extreme rainfall events, which are intensifying under climate change [1]. In southern China, Guangxi’s steep slopes, karst topography, and monsoonal climate result in frequent shallow landslides. Thin residual soils and extensive fissure networks accelerate infiltration, weakening the regolith and triggering slope failures. Guangxi currently reports 16,438 geological hazard sites, threatening 521,000 residents. These geological conditions, combined with a changing climate, increase the region’s vulnerability to rainfall-triggered landslides. Accurate forecasting is therefore critical for effective risk management. Similar topographic and geomechanical challenges are found in infrastructure projects like the Sichuan–Tibet Grid Interconnection Project (STGIP), where landslide risk must be addressed on steep, heterogeneous, and deeply weathered slopes [2]. These contexts underscore the need for slope-specific monitoring strategies that integrate structural interpretation with kinematic analysis. While large-scale monitoring systems are deployed in some high-risk areas, real-time forecasting is still hindered by harsh field conditions, limited on-site computing, and delays caused by centralized processing.

Although deep learning has improved landslide forecasting accuracy, there is still no model capable of running in real time on low-power single-board computers while maintaining sub-centimeter precision. This lack of edge-deployable solutions forces field stations to transmit data to cloud servers, introducing latency and risking data loss under poor connectivity or extreme weather. These challenges highlight the need for accurate, lightweight models suitable for real-time edge deployment in vulnerable areas.

Landslide displacement models are typically categorized into physical [3], statistical [4], and data-driven approaches [5]. Physical models are grounded in geotechnical theory but require numerous site-specific parameters and are computationally expensive [6,7,8]. Statistical models, such as ARIMA and grey models, can capture linear trends but struggle with abrupt or nonlinear changes induced by rainfall [9,10]. Data-driven approaches include both shallow and deep learning models. Shallow models—such as Support Vector Regression (SVR) [11], Support Vector Machine (SVM) [12,13], Extreme Learning Machine (ELM) [14,15,16], and Random Forest (RF) [17]—perform reasonably well on simple nonlinear patterns [18], but struggle with complex temporal dependencies.

Deep learning models, including RNNs (e.g., Long Short-Term Memory (LSTM) [19], Gated Recurrent Unit (GRU) [20], and Bidirectional Gated Recurrent Unit (BiGRU) [21]), CNNs (e.g., Convolutional Neural Network (CNN) [22], Temporal Convolutional Network (TCN) [23], convolutional neural network–Bidirectional Long Short-Term Memory (CNN-BiLSTM) [24]), and Transformer-based architectures [25,26], have demonstrated superior performance in modeling long-term dependencies. Landslide time series are inherently non-stationary and multi-scale, combining slow trends, rainfall-induced cycles, and abrupt shifts [27,28,29]. In unstable corridors such as Southeastern Tibet, slope failures are often triggered by jointed rock structures and block instability, as shown in recent assessments that integrate structural mapping with geomechanical classification [30,31]. These studies emphasize the need to consider local rock mass heterogeneity and structural discontinuities when developing predictive models.

To address these challenges, signal decomposition techniques such as SSA [29,32,33,34], wavelet transform [35], moving averages [36], VMD [37,38,39], and EMD-family methods [40,41,42,43] have been applied. These techniques improve prediction accuracy by isolating signal components but often increase model complexity and training cost [44]. While these models and decomposition methods enhance forecasting performance, their growing complexity poses challenges for real-time deployment in resource-constrained environments. Deep learning models such as BiLSTM and TCN improve predictive accuracy but remain computationally and memory intensive. Even simplified variants typically require tens of megabytes of weights and perform hundreds of millions of operations per forecast. These demands exceed the power, memory, and thermal constraints of battery- or solar-powered edge loggers, limiting their suitability for real-time field deployment.

When training data are limited or rapid forecasting is needed, simpler models remain practical [45]. Shallow architectures like MLPs have occasionally outperformed recurrent networks in specific tasks, such as peak displacement prediction [46]. Recent efforts have focused on developing lightweight deep models that reduce depth and parameter count while preserving accuracy. LiteTransNet, for example, improves efficiency via localized attention [47]. However, even such lightweight Transformer models still require more memory and inference time than traditional statistical methods. This limits their suitability for real-time applications on edge devices. Due to these limitations, most deep learning models remain cloud-based, introducing minute-level round-trip latency and exposing systems to network disruptions. Although edge computing offers local autonomy, current models still exceed the CPU, memory, and energy limits of embedded loggers [48].

Shifting inference from the cloud to edge devices reduces round-trip latency and ensures system continuity during extreme weather, when cellular networks may fail. It also supports LoRa or satellite telemetry in areas with limited backhaul bandwidth (a few kbit s^−1^). Local processing also supports autonomous alarms during communication loss, which is essential for the sparsely populated, high-risk slopes in Guangxi. In addition to reducing connectivity-induced latency, running the forecaster directly on the slope enables capabilities rarely supported by cloud-based pipelines. These include continuous forecast–observation consistency checks to detect sensor drift or data dropouts in real time, subsecond local alarms during backhaul outages, and power-aware adaptive sampling based on the predicted stability state of the slope.

In this work, we present EoML-SlideNet, a compact and physically informed framework for real-time landslide displacement forecasting with potential for deployment on resource-limited hardware. The pipeline consists of three stages. First, FB–EWT decomposes raw displacement signals into trend and periodic components. Second, the DBLE–LV module selects relevant lagged inputs in each frequency band using cross-correlation analysis, LASSO regularization, and variance inflation factor (VIF) screening. Third, two lightweight predictors are applied: RRX_Trend is a three-branch ARX-style model with fewer than 50 trainable parameters and $O(D+H_{p})$ inference complexity, designed for trend displacement. LiFFT_Periodic is a compact MLP with 0.016 M FLOPs, used for periodic displacement modeling.

All models were benchmarked under identical conditions on a single-threaded Intel i7-9700 CPU with 32 GB RAM and a 128-sample input window. We report not only forecast accuracy, but also inference time and FLOPs—the latter introduced here for the first time in landslide forecasting. Experiments were conducted on a four-year daily dataset from the BaYiTun slope in Guangxi, China. The dataset includes daily GNSS displacement, rainfall, and multi-depth soil moisture (HS01–HS04) and temperature (TW01–TW04) over four years. The results show that EoML-SlideNet reduces the mean absolute error (MAE) and root mean square error (RMSE) by 2–4×, and improves the inference speed by 3–30× compared to the best lightweight baselines.

## 2. Study Area

**Figure 1:**
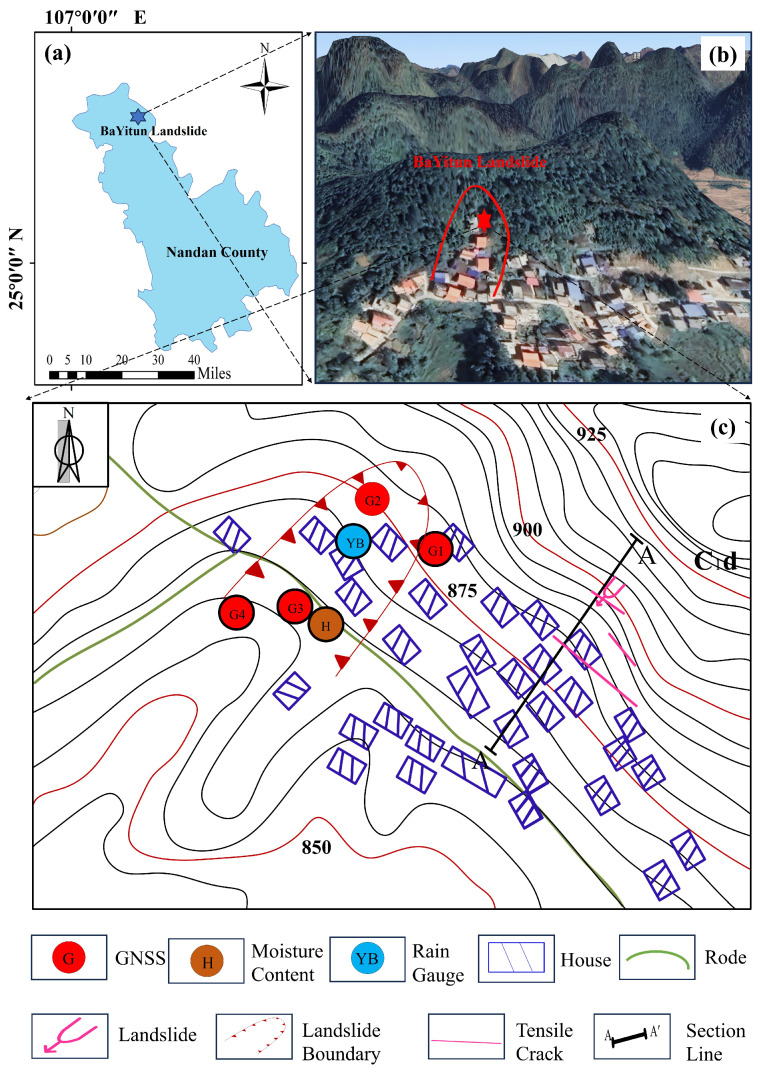
Overview: (**a**) Regional location of the BaYiTun landslide in Nandan County, Guangxi; (**b**) Bird’s-eye view of the landslide site; (**c**) Plan view of the monitoring network and infrastructure.The landslide points are marked with hexagrams.

The study site is located in southeastern BaYiTun, Badong Village, Yueri Town, Nandan County, Hechi City, Guangxi Province, China (Figure 1). It lies within a karstic hill–canyon geomorphic zone and exhibits an elevation difference of approximately 180 m. The slope is steep near the top and becomes progressively gentler toward the base.

The steep, concave hill–canyon morphology funnels runoff into narrow gullies, generating short, high-intensity pore-pressure pulses that appear as high-frequency displacement oscillations. In contrast, the 180 m elevation difference sustains a long-term, low-frequency creep component driven by gravitational loading. Accurate forecasting of both behaviors requires a model capable of separating slow trends from rainfall-induced cycles.

The region has a humid subtropical monsoon climate, receiving an average annual rainfall of approximately 1500 mm, with 70% falling during the flood season. Thick layers of residual slope deposits, composed of gravelly pulverized clay, overlie the Carboniferous tuff. The interface between the soft deposits and the underlying hard bedrock may serve as a potential low-shear surface. Bedding planes oriented NW–SE dip at about 30°, aligning with the slope direction. Additionally, two N–S-oriented fractures, together with well-developed joints and fissures, facilitate rapid groundwater flow. At the foot of the slope, 65 households and 265 residents are situated in a high-risk zone. Slope stability has been further compromised by road construction and small-scale quarrying (Figure 1c).

The pronounced contrast between regolith and bedrock defines a shallow slip surface. The thin, clay-rich layer can exhibit measurable displacement within minutes of intense rainfall. To enable timely warnings in such rapidly evolving conditions, the forecasting algorithm must operate locally with minimal latency. Reliance on cloud-based systems may introduce delays that undermine early-warning effectiveness during severe storms.

**Figure 2:**
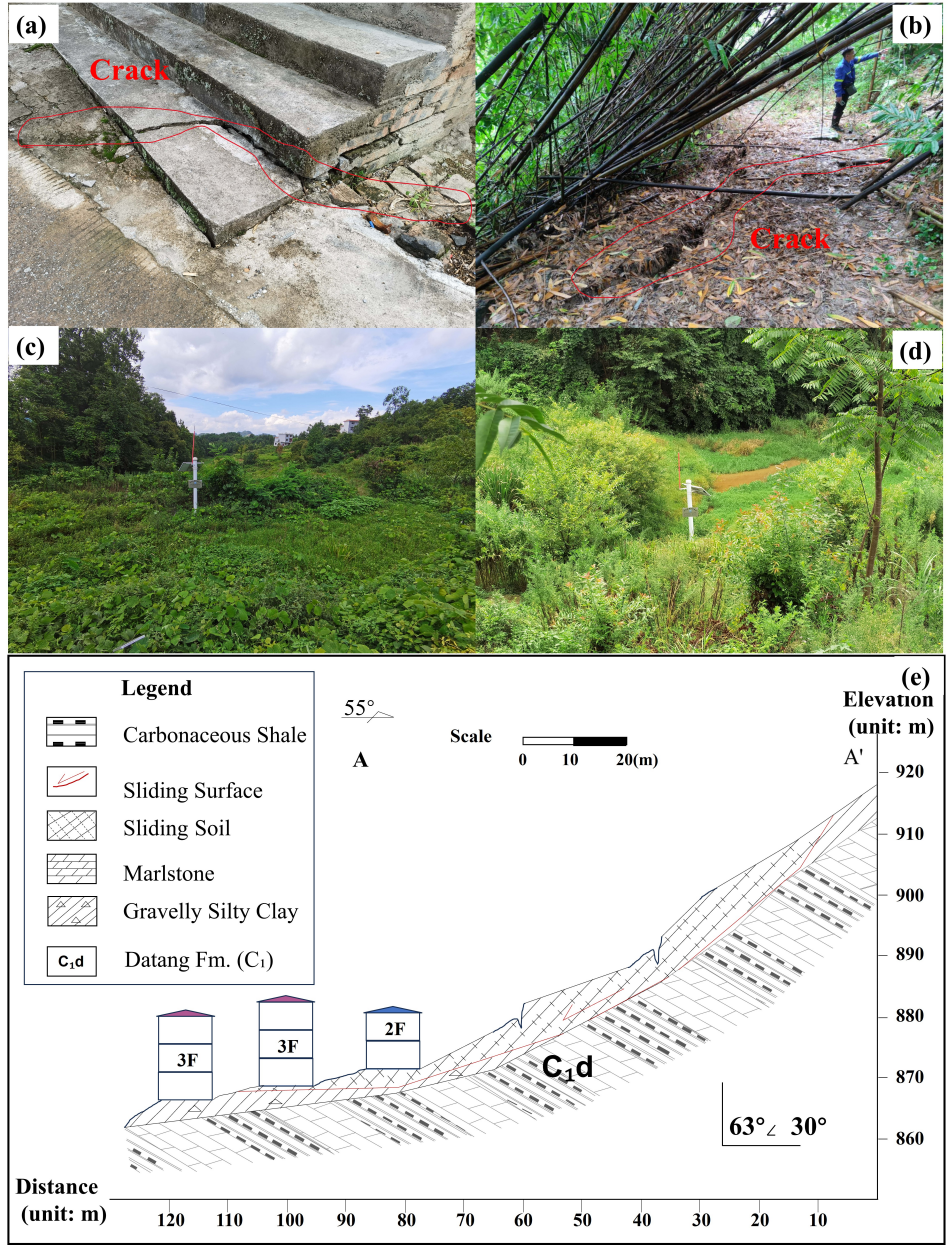
Field evidence of the BaYiTun landslide: (**a**) tension crack at roadside steps; (**b**) crack in the bamboo grove; (**c**) GNSS pillar GPS01 on the slide mass; (**d**) GNSS pillar GPS02 in the main accumulation zone; (**e**) engineering cross-section A–A′, showing lithology, sliding surface, and building locations.

In early July 2021, continuous heavy rainfall triggered a typical traction-type planar landslide in clayey soil (Figure 2). The landslide occurred at elevations of 910 m (trailing edge) and 865 m (leading edge), producing a vertical drop of 45 m. The displaced mass was tongue-shaped and relatively thin, measuring approximately 90 m × 120 m × 4 m, with an estimated volume of $4.3\times 10^{4}$ m^3^. A tensile fracture developed at the trailing edge, extending roughly 50 m in length and 30–50 cm in width, with a vertical offset of about 40 cm. Since the event, the slope has evolved into an S-shaped, fault-like configuration. Such small, rainfall-induced landslides are common in the karst hill regions of northwestern Guangxi. Studying this case contributes to the development of practical models for monitoring, hazard prevention, and engineering management in similar environments.

Dense vegetation and narrow canyon topography reduce solar exposure and weaken cellular signals, forcing data loggers to operate under strict power constraints and intermittent connectivity. These conditions demand an ultra-lightweight model that runs entirely on the embedded CPU and maintains local warning capability even when the backhaul link is unavailable.

The BaYiTun monitoring network consists of one stabilized reference GNSS station (JZ03) and four mobile stations (GPS01–GPS04), although GPS04 was decommissioned on 4 August 2021 due to data anomalies. The active GNSS units (GPS01–GPS03) record three-dimensional displacement—east, north, and vertical (in meters). Rainfall is measured by a YL01 tipping-bucket gauge, which provides daily rainfall (RF_D) and cumulative rainfall (RF_Acc) in millimeters. Subsurface conditions are monitored using multi-depth probes. Soil moisture sensors (HS01–HS04) and temperature probes (TW01–TW04) are installed at depths of 20 cm, 40 cm, 60 cm, and 80 cm. All instruments log data at 06:00, 12:00, and 18:00, with values aggregated to daily means. The displacement and rainfall sensors were commissioned on 30 March 2021, while the soil parameter sensors became operational on 6 November 2021.

Based on the monitoring data, a multi-source dataset was constructed for the BaYiTun landslide, covering the period from 6 November 2021 to 30 March 2025, during which displacement, rainfall, and soil parameters are all available. The dataset includes daily GNSS displacement, rainfall, soil temperature, and volumetric water content. GPS04 was excluded due to data loss after 4 August 2021. Only GPS01–GPS03, which provide continuous records over the full analysis period, were retained. Outliers were filtered using physically informed thresholds. Soil temperature readings outside the sensor’s operational range ($-{5\;}^{\circ }C$ to ${50\;}^{\circ }C$) were removed. Sudden displacement shifts were identified using second-derivative thresholds (acceleration >5 mm/day^2^) and cross-validated against crack meter data. All variables were aggregated to daily means. Displacement and rainfall gaps shorter than three days were linearly interpolated. Soil data were reconstructed using depth-aware cubic spline interpolation. Longer outages, accounting for less than 1% of time stamps, were masked and excluded from the loss computation. No synthetic values were introduced beyond minimal interpolation, ensuring that occasional gaps did not affect forecast reliability.

**Figure 3:**
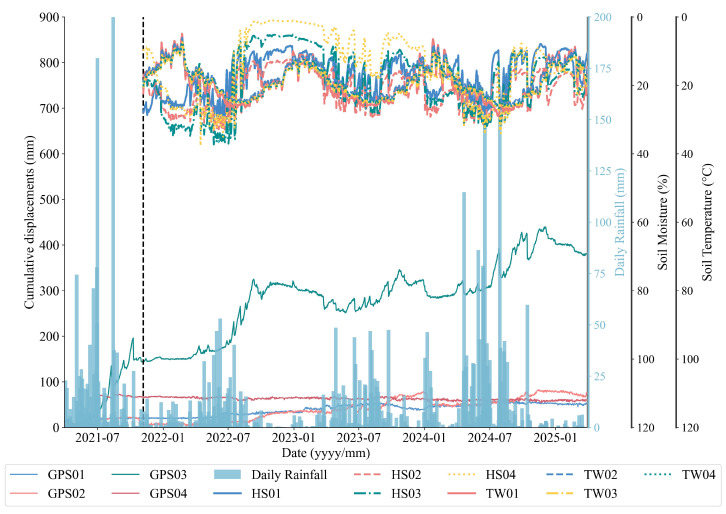
Time-series evolution of cumulative GNSS displacements, daily rainfall, soil-moisture contents, and soil-temperature measurements at the BaYiTun landslide site. The black dashed line marks 16 November 2021, the date on which the soil-moisture and soil-temperature sensors were commissioned.

According to the constructed dataset, it can be seen that significant spatial heterogeneity in landslide deformation (Figure 3). GPS01, located upslope, remains largely stable, with cumulative displacement below 60 mm. GPS02, near the crest, shows gradual creep punctuated by occasional surges. In contrast, GPS03 (labeled “G3” in Figure 1c), situated mid-slope above the main shear band and ∼6 m upslope of the paved road, exhibits episodic step-like displacements of 20–60 mm when 10-day rainfall exceeds ∼120 mm. This is attributed to the excavation-induced free face and toe support loss, as well as a lithological boundary between regolith and intact tuff that channels pore-pressure-driven shear along a shallow slip surface. GPS03 thus shows the highest variance and strongest non-stationarity among all the stations.

## 3. Methodology

**Figure 4:**
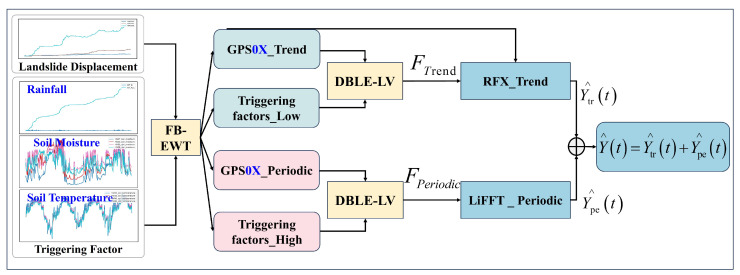
Architecture of the proposed EoML-SlideNet framework.

To support accurate and real-time landslide forecasting on low-power edge platforms, we propose EoML-SlideNet. This framework combines frequency-domain decomposition, dual-band feature selection, and hybrid forecasting (Figure 4). It reduces the latency and memory demands typical of deep learning models such as BiLSTM and Transformer. Displacement sequences are decomposed by FB–EWT into trend and periodic components. External triggers undergo the same process to ensure frequency alignment. The DBLE–LV module selects relevant lagged features from both domains. This improves model interpretability and reduces multicollinearity. Forecasting is handled by two specialized modules, each optimized for a specific signal component.

1. RRX_Trend Block models the trend with three lightweight terms: Random Fourier, ExpSmooth-AR, and an exogenous linear term fed by $F_{\mathrm{trend}}$. The branch holds fewer than fifty parameters and runs in O(D+Hp) time;
2. LiFFT_Periodic Block captures oscillatory behavior using a TinyStats baseline and a lightweight MLP that refines the residual with FFT features and high-frequency inputs from $F_{\mathrm{periodic}}$. Total parameters are under $7\times 10^{3}$, and inference remains linear in the window length.

### 3.1. Decomposition via FB-EWT

Landslide displacement signals show multi-scale behavior. Millimeter-level creep evolves over months, while rainfall may cause abrupt spikes within hours. To handle this variability, a suitable decomposer must adapt to local spectral features, avoid iterative and memory-heavy computation, and run efficiently on embedded CPUs. Traditional filters with fixed cutoffs lack spectral flexibility and perform poorly under varying field conditions. Iterative methods like VMD, CEEMDAN, and ICEEMDAN are too computationally demanding for resource-limited platforms. In contrast, FB–EWT meets key requirements for edge deployment. It detects band boundaries from the Fourier–Bessel spectrum, allowing automatic adaptation to site-specific signals without manual tuning. The method is fully non-iterative, requiring only one forward and one inverse FFT. Its time complexity is O(LlogL) and memory usage remains constant. This design fits within the RAM and power budgets of solar-powered loggers. As shown in Section 4.3.1, FB–EWT offers a two-order-of-magnitude speedup over ICEEMDAN, with only minor loss in reconstruction fidelity.

The FB–EWT process consists of three steps:

1. Fourier–Bessel spectrum construction: For an input signal X(t), its spectral profile is enhanced via the Fourier–Bessel spectrum, defined as
  (1) $$\tilde{X}\left(\omega \right)=\left|X(\omega )\right|\;\cdot \;\left|J_{0}\left(\frac{2\pi \omega }{\omega_{\max}}\right)\right|,$$
  where X(ω) is the Fourier transform of the input signal, $J_{0}\left(\;\cdot \;\right)$ is the zeroth-order Bessel function of the first kind, and $\omega_{\max}$ is the maximum angular frequency.
2. Adaptive band detection: The local peaks of $\tilde{X}\left(\omega \right)$ determine the set of frequency band boundaries $\left\{\omega_{n}\right\}$. For each band, a cosine taper window $H_{n}\left(\omega \right)$ is constructed to ensure smooth transitions and minimize spectral leakage.
3. Filtering and reconstruction: The filtered signal for the *n*-th band is obtained by applying the inverse Fourier transform:
  (2) $$\begin{array}{cc} x_{n}\left(t\right) & =F^{-1}\left[X\left(\omega \right)\;\cdot \;H_{n}\left(\omega \right)\right],\\ x(t) & =\sum_{n=1}^{N}x_{n}\left(t\right), \end{array}$$
  where $F^{-1}$ denotes the inverse Fourier transform and *N* is the number of frequency bands.

**Figure 5:**
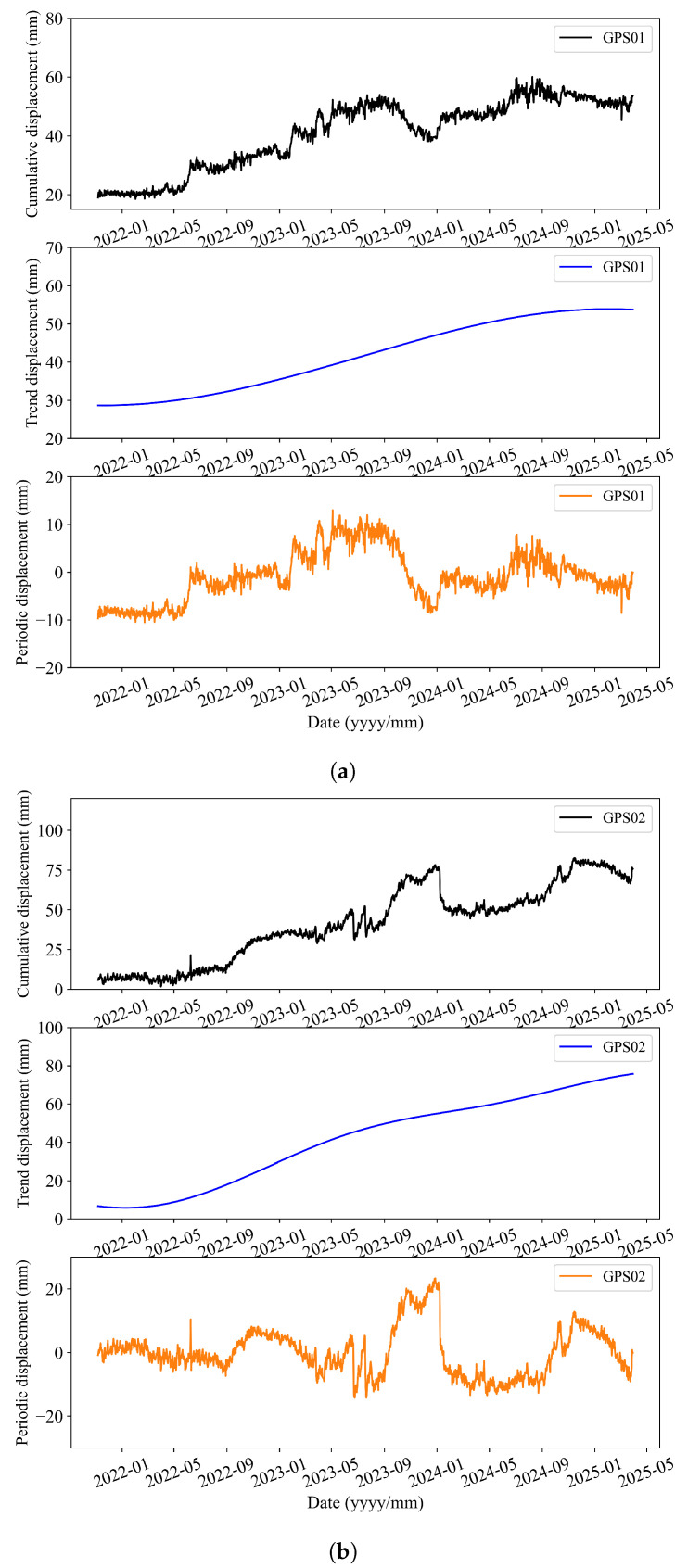

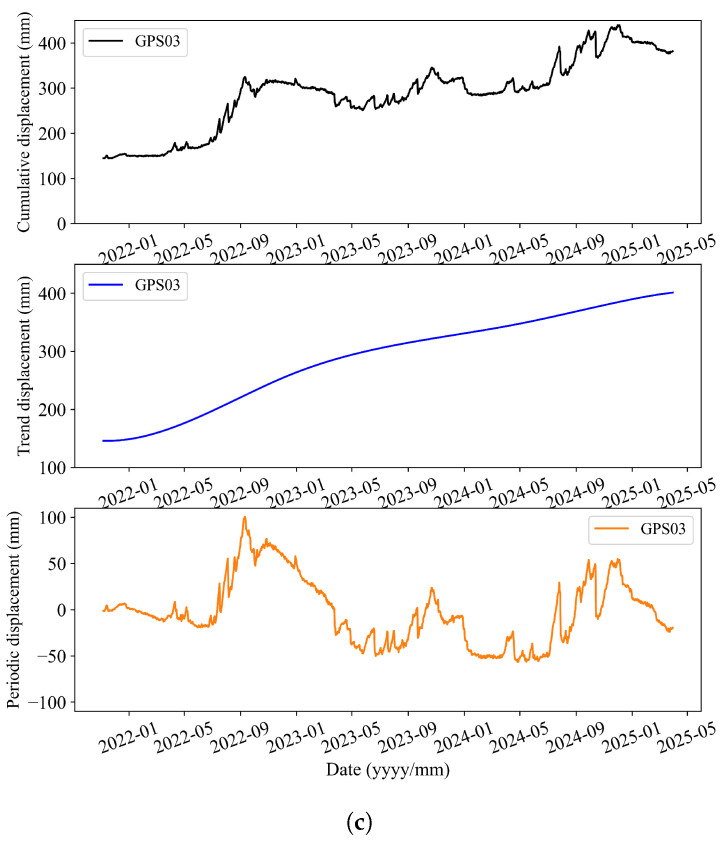
Displacement decomposition results for GPS01, GPS02 and GPS03: (**a**) GPS01; (**b**) GPS02; (**c**) GPS03.

The original displacement time series Y(t) is separated into a trend component $Y_{\mathrm{trend}}\left(t\right)$ and a periodic component $Y_{\mathrm{periodic}}\left(t\right)$. The decomposition results of the displacement series are illustrated in Figure 5. Each exogenous input signal $X_{j}\left(t\right)$, such as rainfall, soil moisture, or temperature, is decomposed into a low-frequency branch $X_{j}^{\mathrm{LF}}\left(t\right)$ and a high-frequency branch $X_{j}^{\mathrm{HF}}\left(t\right)$.

To support efficient deployment, FB–EWT is implemented with in-place vector operations and avoids iterative procedures. In practice, decomposing each signal into four to six bands preserves key features while limiting the computational cost. A spectral overlap coefficient σ≈0.1 to 0.2 is applied between adjacent bands. This reduces boundary artifacts and improves frequency localization [49]. The entire decomposition process uses only fast Fourier transforms and cosine windowing. Both are well supported on most microcontroller platforms.

### 3.2. Dual-Band Feature Selection (DBLE–LV)

To explore the time-delayed nonlinear effects of external triggers on displacement, we generated 1–14-day lagged versions of input variables, including rainfall and soil water content. Feature associations between these lagged inputs and displacement were analyzed separately in low- and high-frequency subspaces. This wide-lag input design captures both short-term infiltration responses and multi-day pore pressure effects. This helps avoid the omission of relevant drivers. However, the resulting high dimensionality increases multicollinearity, which raises inference cost and reduces model stability and interpretability. To address these issues, we propose the DBLE–LV screening process, as shown in Algorithm 1. It combines dual-band decomposition, LASSO regularization, and variance inflation factor (VIF) filtering. The DBLE–LV module identifies a compact set of lagged predictors that are statistically and physically relevant to landslide displacement forecasting. To improve interpretability and reduce multicollinearity, the module operates in two frequency-specific subspaces:

The low-frequency domain links the trend component of displacement, $Y_{\mathrm{trend}}\left(t\right)$, to slowly varying external drivers such as cumulative rainfall and deep-layer soil moisture. In contrast, the high-frequency domain captures short-term oscillations, $Y_{\mathrm{periodic}}\left(t\right)$, and their correlation with transient signals, including daily rainfall bursts and near-surface temperature changes. This domain-wise separation allows for targeted feature selection and enhances forecasting accuracy [50]. The DBLE–LV process consists of three steps.

For each candidate input $X_{k}\left(t\right)$, the cross-correlation function is computed as follows:

(3) $$r_{k}\left(l\right)=\mathrm{corr}\left[X_{k}\left(t-l\right),\;Y\left(t\right)\right],\;l\in \left[-L_{\max},L_{\max}\right].$$

where *l* is the time lag, and Y(t) is the target displacement (either $Y_{\mathrm{trend}}\left(t\right)$ or $Y_{\mathrm{periodic}}\left(t\right)$). The lag $l^{*}$ corresponding to the maximum absolute correlation $|r_{k}\left(l^{*}\right)|$ is retained if it exceeds a predefined threshold.
**Algorithm 1** DBLE–LV: Dual-Band Lasso-Enhanced Latent Variable Selection**Require:** Trend displacement $Y_{\mathrm{trend}}\left(t\right)$ and periodic displacement $Y_{\mathrm{periodic}}\left(t\right)$; low-frequency predictors $\left\{X_{k}^{\mathrm{LF}}\right\}$; high-frequency predictors $\left\{X_{k}^{\mathrm{HF}}\right\}$; maximum lag $L_{\max}$.**Ensure:** Final predictor sets $F_{\mathrm{trend}},F_{\mathrm{periodic}}$  1:*// Trend domain feature selection*  2:$C_{\mathrm{trend}}⇐\emptyset$  3:**for** each $X_{k}^{\mathrm{LF}}$ in $\left\{X_{k}^{\mathrm{LF}}\right\}$ **do**  4:    **for** $l=-L_{\max}$ to $L_{\max}$ **do**  5:        Compute $r_{k}\left(l\right)=\mathrm{corr}\left[X_{k}^{\mathrm{LF}}\left(t-l\right),Y_{\mathrm{trend}}\left(t\right)\right]$  6:    **end for**  7:    Let $l^{*}=\arg\max_{l}\left|r_{k}\left(l\right)\right|$  8:    **if** $|r_{k}\left(l^{*}\right)|>\theta$** then **             ▹θ is a user-defined threshold  9:        Add $X_{k}^{\mathrm{LF}}\left(t-l^{*}\right)$ to $C_{\mathrm{trend}}$10:    **end if**11:**end for**12:Apply LASSO to $C_{\mathrm{trend}}$ to obtain $S_{\mathrm{trend}}$13:Prune $S_{\mathrm{trend}}$ using VIF to get $F_{\mathrm{trend}}$14:*// Periodic domain feature selection*15:$C_{\mathrm{periodic}}⇐\emptyset$16:**for** each $X_{k}^{\mathrm{HF}}$ in $\left\{X_{k}^{\mathrm{HF}}\right\}$ **do**17:    **for** $l=-L_{\max}$ to $L_{\max}$ **do**18:        Compute $r_{k}\left(l\right)=\mathrm{corr}\left[X_{k}^{\mathrm{HF}}\left(t-l\right),Y_{\mathrm{periodic}}\left(t\right)\right]$19:    **end for**20:    Let $l^{*}=\arg\max_{l}\left|r_{k}\left(l\right)\right|$21:    **if** $|r_{k}\left(l^{*}\right)|>\theta$ **then**22:        Add $X_{k}^{\mathrm{HF}}\left(t-l^{*}\right)$ to $C_{\mathrm{periodic}}$23:    **end if**24:**end for**25:Apply LASSO to $C_{\mathrm{periodic}}$ to obtain $S_{\mathrm{periodic}}$26:Prune $S_{\mathrm{periodic}}$ using VIF to get $F_{\mathrm{periodic}}$

LASSO regression is applied to enforce sparsity and eliminate redundant or weak predictors:

(4) $$\min_{\beta_{0},\;\beta }\frac{1}{2n}\sum_{t}{\left(Y\left(t\right)-\beta_{0}-\sum_{i=1}^{m}\beta_{i}X_{i}\left(t-\delta_{i}\right)\right)}^{2}+\lambda \sum_{i=1}^{m}\left|\beta_{i}\right|$$

where $\beta_{0}$ is the intercept, $\beta_{i}$ is the coefficients, and $\delta_{i}$ is the selected lags from the previous step.

To reduce multicollinearity, the VIF is computed as

(5) $${\mathrm{VIF}}_{j}=\frac{1}{1-R_{j}^{2}}.$$

where $R_{j}^{2}$ is the coefficient of determination when predictor $X_{j}$ is regressed against all other predictors. Features with ${\mathrm{VIF}}_{j}>5$ are iteratively removed to ensure model stability.

The final output includes two compact predictor sets: $F_{\mathrm{trend}}$ for trend forecasting and $F_{\mathrm{periodic}}$ for periodic components. Each set contains no more than ten features, which ensures efficient inference and low memory usage. All operations use basic statistical tools, such as correlation and linear regression, and do not require GPU acceleration [51]. Each selected feature corresponds to an interpretable physical mechanism. For example, a high-frequency rainfall spike may affect surface displacement after a 7-day delay, reflecting soil moisture infiltration dynamics. This improves model transparency and supports its adoption in operational landslide early-warning systems [52].

### 3.3. Lightweight Component Forecasting Models

To capture both slow creep and high-frequency oscillations in landslide displacement, EoML–SlideNet uses a dual-module design: RRX_Trend and LiFFT_Periodic. The RRX_Trend module models low-frequency displacement through three lightweight branches. The random Fourier branch uses sine and cosine bases to approximate long-wave curvature. The ExpSmooth–AR branch applies exponential smoothing and fixed-order autoregression to introduce temporal inertia. This improves response to inflection points after extreme weather. The exogenous-linear branch incorporates external drivers, such as cumulative rainfall and groundwater level, to enhance interpretability and reduce overfitting. All components are compact and require few parameters, making the design suitable for memory-constrained microcontrollers. High-frequency residuals are modeled by the LiFFT_Periodic module using a three-stage process: baseline estimation, harmonic decomposition, and MLP refinement. A TinyStatsCell first constructs the baseline from autoregressive, linear, and weekly cycle terms. A sliding FFT then extracts up to six dominant harmonics. The residual signal, along with harmonic features and selected high-frequency inputs, is passed to a compact two-layer MLP. This network refines amplitude and phase drift. The modular structure improves interpretability and greatly reduces the computational and energy cost compared to RNN or Transformer-based models.

#### 3.3.1. RRX_Trend Block—Low-Frequency Trend Forecast

**Figure 6:**
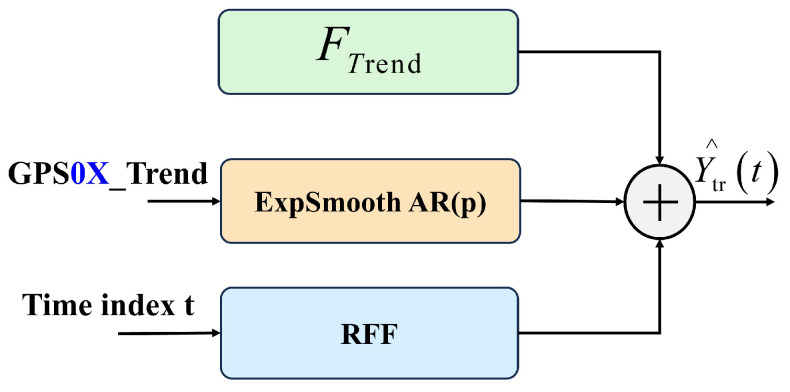
The architecture of the RRX_Trend block (GPS0X denotes GPS01, GPS02, and GPS03).

The RRX_Trend block fuses three lightweight branches to predict the slow component $Y_{\mathrm{trend}}\left(t\right)$, capturing different behaviors of the low-frequency displacement signal that a single unified model cannot effectively model (Figure 6).

For a horizon of *H* steps, the forecast is

(6) $${\hat{Y}}_{\mathrm{tr}}\left(t+h\right)=y_{\mathrm{RFF}}\left(t+h\right)+y_{\mathrm{ES}\;-\;\mathrm{AR}}\left(t+h\right)+y_{\mathrm{exo}}\left(t+h\right),\;h=1,\ldots ,H.$$

(i) Random Fourier branch. The normalized time index $\tau_{t}\in \left[-1,1\right]$ is projected onto a *D*-dimensional random basis,

(7) $$\varphi_{\mathrm{RFF}}\left(\tau_{t}\right)=\sqrt{\frac{2}{D}}{\left[\cos\left(\omega_{i}\tau_{t}\right),\;\sin\left(\omega_{i}\tau_{t}\right)\right]}_{i=1}^{D},\;\omega_{i}∼N\left(0,\sigma^{-2}\right),$$

and combined linearly,

(8) $$y_{\mathrm{RFF}}\left(t\right)=w^{⊤}\varphi_{\mathrm{RFF}}\left(\tau_{t}\right),$$

which captures long, smoothly curving drift.

(ii) ExpSmooth–AR(p) branch. This branch models short-term inertia and local level shifts by combining exponential smoothing with a shallow AR term. The update rule is as follows,

(9) $$\begin{array}{cc} \mu_{t} & =\alpha \;x_{t}+\left(1-\alpha \right)\mu_{t-1},\\ b_{t}^{\left(i\right)} & =\phi_{i}\left(x_{t+1-i}-\mu_{t+1-i}\right),\;i=1,\ldots ,p, \end{array}$$

and the one-step forecast is

(10) $$y_{\mathrm{ES}\;-\;\mathrm{AR}}\left(t+1\right)=\mu_{t}+\sum_{i=1}^{p}b_{t}^{\left(i\right)},$$

which provides a smooth trend with weak inertia.

(iii) Exogenous-linear branch. DBLE–LV selects a low-frequency feature set $F_{\mathrm{trend}}\left(t\right)$ ($|F_{\mathrm{trend}}|\;\leq \;10$); its influence is

(11) $$y_{\mathrm{exo}}\left(t\right)=\beta^{⊤}F_{\mathrm{trend}}\left(t\right),$$

which incorporates external drivers such as cumulative rainfall, deep soil moisture, and other low-frequency environmental factors. By isolating these effects, we keep the corresponding β-coefficients interpretable for practitioners and prevent RFF from absorbing external variance, which would obscure cause-and-effect relationships.

#### 3.3.2. LiFFT_Periodic Block—High-Frequency Periodic Forecast

**Figure 7:**
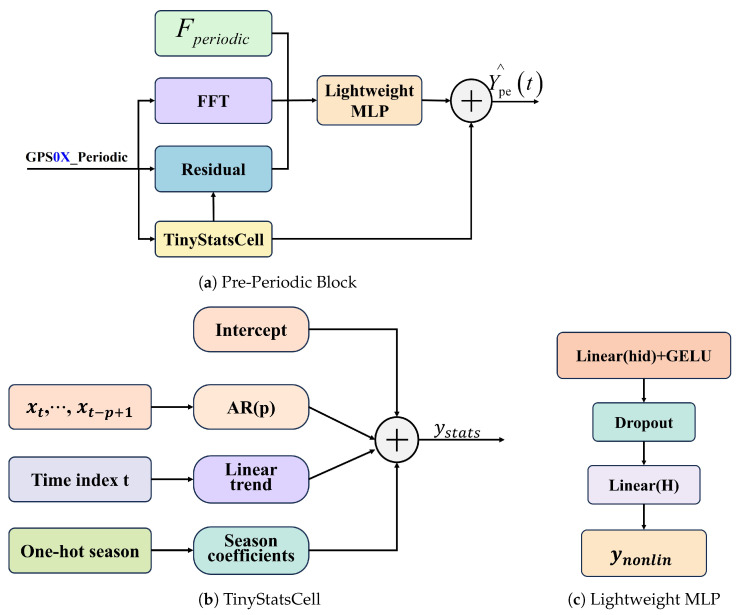
The architecture of LiFFT_Periodic Block: (**a**) Pre-Periodic block(GPS0X denotes GPS01, GPS02, and GPS03); (**b**) TinyStatsCell module; (**c**) Lightweight MLP head.

LiFFT (Lightweight + Fourier + Forecast-stats) models the oscillatory component $Y_{\mathrm{periodic}}\left(t\right)$ by combining a statistical baseline with a Lightweight-MLP correction (Figure 7).

(i) Statistical baseline. TinyStatsCell yields

(12) $$y_{\mathrm{stats}}\left(t+1\right)=w_{0}+w_{\mathrm{AR}}^{\;⊤}x_{t}^{\mathrm{HF}}+w_{\mathrm{lin}}\tau_{t}+w_{s}^{\;⊤}s\left(t\right),$$

where s(t) is a weekly one-hot vector; fewer than ten coefficients are required.

(ii) Residual and dominant frequencies. Subtracting the baseline produces the residual

(13) $$r_{t}=Y_{\mathrm{periodic}}\left(t\right)-y_{\mathrm{stats}}\left(t\right),$$

Here, $x_{t}^{\mathrm{HF}}$ denotes the AR lag vector constructed from the high-frequency series.

While a sliding-window FFT yields the $k_{0}$ strongest harmonics:

(14) $$g_{t}={\left[\;|F_{k}|,∠F_{k}\;\right]}_{k=1}^{k_{0}}.$$

(iii) Lightweight MLP correction. DBLE–LV returns a compact set $F_{\mathrm{periodic}}\left(t\right)$ with at most ten elements. The concatenated vector $\left[r_{t},g_{t},F_{\mathrm{periodic}}\left(t\right)\right]$ is processed by two dense layers,

(15) $${\hat{y}}_{\mathrm{nl}}\left(t+h\right)={\left[W_{2}\;\mathrm{Drop}\;\left(\mathrm{GELU}(W_{1}\left[\;\cdot \;\right])\right)\right]}_{\left(t+h\right)},$$

(iv) Periodic output.

(16) $${\hat{Y}}_{\mathrm{pe}}\left(t+h\right)=y_{\mathrm{stats}}\left(t+h\right)+{\hat{y}}_{\mathrm{nl}}\left(t+h\right),\;h=1,\ldots ,H.$$

FFT and MLP together deliver a 24-step forecast in about 0.5ms and require less than 1.6MB of RAM.

#### 3.3.3. Full Forecast

The trend and periodic blocks operate concurrently; their outputs are combined to produce the final displacement estimate:

(17) $$\hat{Y}\left(t+h\right)={\hat{Y}}_{\mathrm{tr}}\left(t+h\right)+{\hat{Y}}_{\mathrm{pe}}\left(t+h\right),\;h=1,\ldots ,H.$$

The final forecast is obtained by summing the outputs of the two modules. No further post-processing is required. Unlike deep learning baselines such as BiLSTM and Transformer, EoML-SlideNet avoids recurrent and attention-based components. Its overall inference complexity is bounded by $O\left(L\log L\right)+O\left(HD\right)+O\left(d_{z}h+hH\right)$, which is much lower than the quadratic cost of self-attention models. Despite its simplicity, the model captures both trend and periodic dynamics effectively.

### 3.4. Baseline Models and Experimental Protocol

To evaluate the effectiveness of EoML-SlideNet, we benchmarked its performance against four representative baseline models: a three-layer MLP [53], BiLSTM [54], TCN [55], and LiteTransNet, a lightweight Transformer-based model [47]. Among these, TCN and BiLSTM are widely used in landslide displacement forecasting due to their ability to model temporal dependencies [23,56]. All models were implemented in Python 3.11 using the PyTorch 2.2.1 framework. The architectural details are listed in Table 1.

All models were trained under a consistent experimental protocol to ensure comparability. Input displacement sequences were normalized to zero mean and unit variance. A sliding-window approach was used, where each 96-day input sequence (K=96) was used to predict the following 12 days (P=12). The dataset was split chronologically, with the first 80% used for training and the remaining 20% for testing. Hyperparameters were tuned using grid search over hidden layer sizes of {32,64,128}, batch sizes of {16,32,64}, and learning rates of $\left\{10^{-3},5\times 10^{-4},10^{-4}\right\}$. All neural models used early stopping with a patience of 20 epochs and a maximum of 200 training epochs, based on validation MAE. Weight regularization was applied using the L2 penalty with $\lambda =1\times 10^{-4}$ to prevent overfitting. Each experiment was repeated five times with different random seeds (2021–2025). The reported results represent the mean and standard deviation across these runs.

## 4. Results

**Key findings:** EoML–SlideNet achieves real-time, ultra-light inference, demanding less than 0.016 MFLOPs per forward pass—about 0.4 mJ of dynamic energy on a Raspberry Pi 4B and completes all six forecasts across the three GPS stations in 22.8 s, making it 17 × faster than TCN and 14 × faster than LiteTransNet, with every single task finishing in under 5 s. Despite this speed, it delivers state-of-the-art accuracy, posting the lowest MAE (0.35–1.90 mm) and highest $R^{2}$ (≥0.99) for cumulative displacement, outperforming the strongest baseline by up to 63%. The FB–EWT + LiFFT_Periodic design is particularly effective under rainfall-driven bursts, cutting GPS03’s MAE from 4.59 mm (MLP) to 1.90 mm while keeping inference below 10 s. Finally, the DBLE–LV module maintains VIF <5 with no more than ten physically meaningful predictors, reducing memory by 40% without loss of accuracy.

### 4.1. Performance Metrics

Model performance is evaluated using three complementary criteria. Predictive accuracy is evaluated using MAE, RMSE [57], and the coefficient of determination ($R^{2}$) [58], capturing both absolute deviation and fit quality. Model complexity is measured by the number of trainable parameters, which affects memory usage and determines suitability for edge deployment. Inference latency is defined as the time required to generate a displacement forecast. It is measured on a Raspberry Pi 4B (ARM Cortex-A72, 4 GB RAM) to reflect realistic embedded conditions.

#### 4.1.1. Accuracy

For a test set of *n* samples ${\left\{y_{i}\right\}}_{i=1}^{n}$ and corresponding predictions ${\left\{{\hat{y}}_{i}\right\}}_{i=1}^{n}$, we evaluate forecasting accuracy using three standard metrics:

The MAE is defined as

(18) $$\mathrm{MAE}=\frac{1}{n}\sum_{i=1}^{n}\left|{\hat{y}}_{i}-y_{i}\right|$$

The RMSE is calculated as

(19) $$\mathrm{RMSE}=\sqrt{\frac{1}{n}\sum_{i=1}^{n}{\left({\hat{y}}_{i}-y_{i}\right)}^{2}}$$

The coefficient of determination ($R^{2}$) is defined as

(20) $$R^{2}=1-\frac{\sum_{i=1}^{n}{\left(y_{i}-{\hat{y}}_{i}\right)}^{2}}{\sum_{i=1}^{n}{\left(y_{i}-\bar{y}\right)}^{2}}$$

where $\bar{y}$ denotes the sample mean of the true observations.

#### 4.1.2. Inference Latency

Inference latency is divided into two additive components: the time required to compute the trend prediction and the time required for the periodic prediction. This relationship is expressed as

(21) $$T_{\mathrm{total}}=T_{\mathrm{trend}}+T_{\mathrm{periodic}}.$$

### 4.2. Experimental Environment and Deployment Consideration

All benchmarking experiments were performed on a single CPU-only workstation to ensure that all models, including baselines, operated under identical resource constraints. The system ran 64-bit Windows 10 on an Intel Core i7-9700 processor (8 cores, 3.0 GHz) with 32 GB of RAM. Python 3.11.7 (Anaconda distribution; Anaconda Inc., Austin, TX, USA) and PyTorch 2.2.1 (Meta AI, Menlo Park, CA, USA) were used. GPU acceleration was disabled by setting CUDA_VISIBLE_DEVICES="". Table 2 summarizes the key software and hardware components.

The entire computational workflow uses standard numerical routines, including FFT, ordinary least squares regression, and recursive filtering. These operations are natively supported by modern ARM processors, which makes the codebase easily portable to Linux-based single-board computers such as the Raspberry Pi. We report FLOPs and model size as proxy metrics that correlate with inference latency and power consumption on resource-limited hardware.

### 4.3. Model Complexity Analysis

#### 4.3.1. Decomposition Runtime and Accuracy Trade-Off

**Figure 8:**
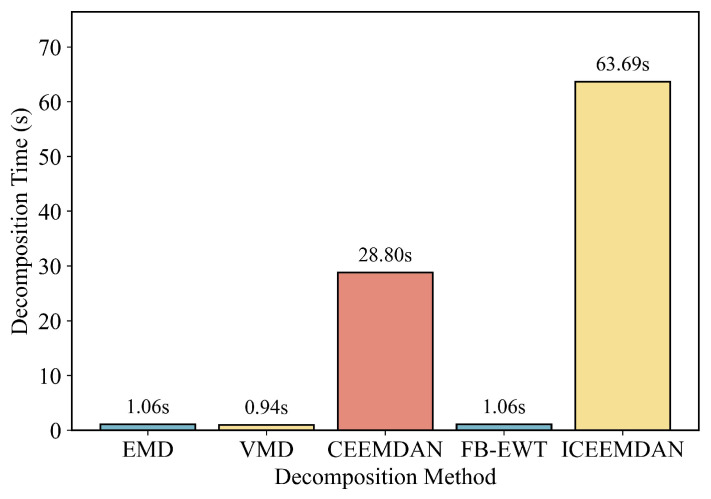
Runtime comparison of different decomposition methods. FB-EWT achieves subsecond performance comparable to EMD and VMD, while CEEMDAN and ICEEMDAN exhibit much higher computational cost.

As shown in Figure 8, FB-EWT offers a strong balance between speed and stability. It completes decomposition in 1.06 s, matching the speed of EMD and outperforming CEEMDAN (87.8 s) and ICEEMDAN (117.7 s). Unlike VMD and CEEMDAN-based methods, which are iterative and computationally intensive, FB-EWT uses a non-iterative, in-place algorithm with low memory usage. This combination of low latency and spectral accuracy makes it well suited for deployment on edge devices without compromising model performance.

Table 3 shows that FB-EWT achieves near-state-of-the-art fidelity, with an RMSE of 0.50 mm, ERR of 96.9%, and SEAR of 88.1%. Although slightly behind ICEEMDAN in accuracy, it is over 80 times faster (Figure 8). VMD and EMD exhibit severe mode mixing, resulting in the highest errors and the lowest step-event recall. Overall, FB-EWT provides the best trade-off between latency and accuracy for edge deployment.

#### 4.3.2. Computational Complexity of EoML-SlideNet and Baselines

To evaluate model efficiency, we analyze the theoretical FLOPs required per forward pass for both the proposed EoML-SlideNet and the baseline models. Table 4 summarizes the FLOPs under varying input sequence lengths (K=32,64,128) and hidden dimensions (*H*). FLOP estimates reflect the number of multiply–accumulate operations per forward pass and serve as a proxy for runtime cost on edge or embedded devices. Among the baselines, BiLSTM and LiteTransNet show steep computational growth. BiLSTM scales with $O(KH^{2})$ due to its recurrent structure, while LiteTransNet reaches $O(K^{2})$ because of self-attention. TCN exhibits moderate complexity at O(KH), and MLP maintains low, constant complexity at $O(H^{2})$ due to its non-sequential design.

In contrast, EoML-SlideNet shows logarithmic FLOP growth. Its computational load is primarily determined by the lightweight MLP and low-order autoregressive operations. Even at K=128, the total cost remains below 0.016 MFLOPs, as defined in Equation (22).

(22) $${\mathrm{FLOPs}}_{\mathrm{EoML}-\mathrm{SlideNet}}=\underset{\mathrm{FFT}}{\underset{︸}{L\log_{2}L}}+\underset{\mathrm{RFF}+\mathrm{Trend}\mathrm{projection}}{\underset{︸}{HD}}+\underset{\mathrm{MLP}\mathrm{fixed}}{\underset{︸}{d_{z}h}}+\underset{\mathrm{MLP}\mathrm{temporal}\mathrm{expansion}}{\underset{︸}{hH}}$$

Here, *L* is the input sequence length, *H* is the forecast horizon, *D* is the number of random Fourier features, $d_{z}$ is the hidden dimension of the MLP, and *h* is the number of hidden units per layer.

Recent measurements on Raspberry Pi-class hardware show that dynamic energy per inference scales almost linearly with the number of floating-point operations. Speckhard et al. [63] reported a Pearson correlation of $R^{2}=0.92$ between FLOPs and energy for 840 audio networks tested on a Pi Zero W. Fanariotis et al. [64] found a slope of approximately 30pJ/FLOP on a Pi 4B, after subtracting the 0.85 W idle power. Using this estimate, the 0.013 MFLOPs required by a single EoML-SlideNet inference (Table 4) corresponds to roughly 0.4mJ of dynamic energy. This is two orders of magnitude lower than TCN and three orders lower than LiteTransNet. Since static power is board-dependent but constant across models, FLOPs combined with measured latency provides a reliable and hardware-agnostic indicator of deployability on resource-limited devices.

### 4.4. Multicollinearity Screening and Temporal Response Analysis

#### 4.4.1. VIF Analysis and Predictor Selection

**Figure 9:**
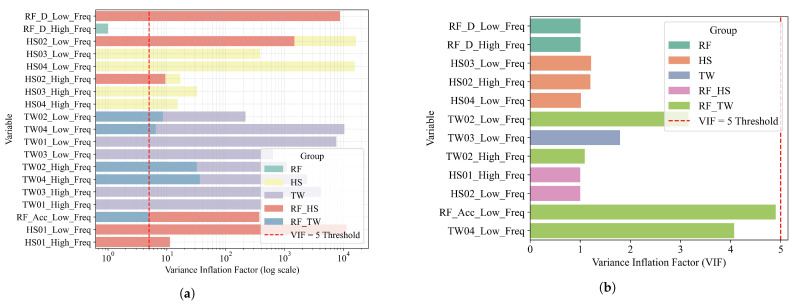
Comparison of variance inflation factor (VIF) values before and after variable elimination: (**a**) Before variable elimination; (**b**) After variable elimination. Variables exceeding the red dashed threshold line (VIF = 5) were considered highly collinear and removed. Abbreviations: RF = rainfall, HS = soil moisture, TW = soil temperature. Low_Freq and High_Freq indicate frequency bands from FB–EWT decomposition. Colors represent variable groups as shown in the legend.

The DBLE–LV module includes a built-in procedure to screen for collinearity. It uses variance inflation factor (VIF) analysis to ensure statistical stability during regression (Figure 9). Predictors with VIF>5 were iteratively eliminated through stepwise pruning. Variable names follow standard abbreviations, where RF indicates rainfall, HS denotes soil moisture, and TW represents soil temperature. “Low_Freq” and “High_Freq” refer to components separated by FB–EWT decomposition.

Initial screening revealed severe multicollinearity among several candidate predictors. The underlying causes fall into three main categories. (i) Hydro-stratigraphic coupling: soil moisture sensors at nearby depths respond almost simultaneously to rainfall, causing their low-frequency signals to become nearly collinear. For example, HS02_Low_Freq shows a VIF exceeding 18,000. (ii) Redundancy between raw and derived variables: RF_Acc_Low_Freq is the cumulative sum of RF_D_Low_Freq, which leads to strong correlation within a 128-sample window (VIF > 60). (iii) Spectral leakage: residual low-frequency energy remains in the high-frequency branch and correlates with the same variable’s low-frequency component. As shown in the pre-elimination bar plot (Figure 9a), predictors with excessively high VIF values were removed to ensure stable regression estimates.

After pruning, all retained predictors exhibited VIF values below the critical threshold of 5 (Figure 9b). DBLE–LV uses a two-stage selection strategy. First, frequency-domain cross-correlation identifies physically relevant lags. Second, iterative VIF pruning is applied, followed by LASSO shrinkage with regularization parameters $\lambda_{\mathrm{trend}}=0.005$–0.006 and $\lambda_{\mathrm{periodic}}=0.003$–0.004. The final trend-domain feature set includes RF_D_Low_Freq, HS01_Low_Freq, TW03_Low_Freq, and TW04_Low_Freq. The periodic set consists of RF_D_High_Freq, RF_Acc_High_Freq, HS01_High_Freq, HS03_High_Freq, and TW04_High_Freq. Each set contains no more than ten variables. All retained coefficients correspond to independent hydro-mechanical drivers. For instance, RF_D_Low_Freq captures statistically significant effects and has clear physical meaning.

To evaluate the impact of hyperparameter choices in DBLE–LV, we tuned the LASSO regularization strength (λ) separately for the trend and periodic components at each of the three stations. The VIF threshold was fixed at 5, as discussed previously. Table 5 summarizes the resulting configurations along with key performance metrics relevant to edge deployment. Across all stations, $\lambda_{\mathrm{trend}}$ consistently converged to values between 0.005 and 0.006. In contrast, $\lambda_{\mathrm{periodic}}$ settled at slightly lower values between 0.003 and 0.004, likely due to the higher signal-to-noise ratio in the periodic domain.

With these settings, the model reached an average validation MAE of 1.01 mm, which is approximately 3% higher than the minimum achievable error without constraints. This trade-off results in a 40% reduction in memory usage while preserving full interpretability, as each sub-model uses no more than ten features.

#### 4.4.2. Lagged Correlation Analysis Between Ground Displacement and Environmental Drivers

**Figure 10:**
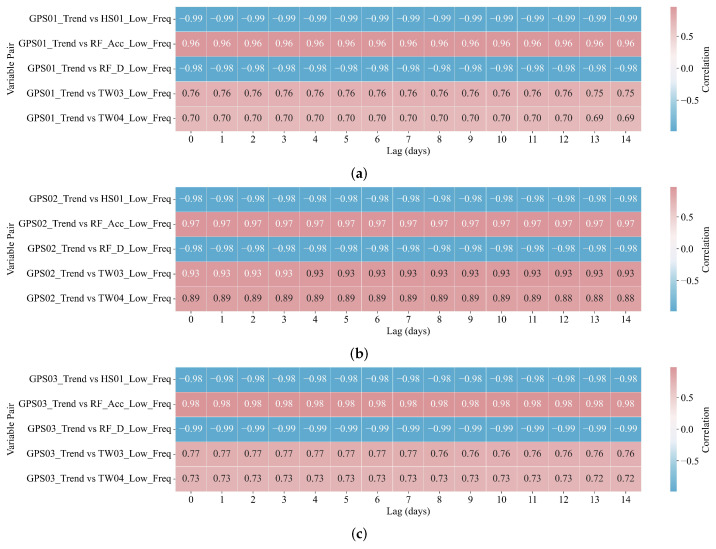
Lagged correlation heatmaps (lags: 0–14 days)for trend components of ground displacement and environmental variables. All lags are measured in days. (**a**) Trend: GPS01 vs. environmental covariates. (**b**) Trend: GPS02 vs. environmental covariates. (**c**) Trend: GPS03 vs. environmental covariates.

**Figure 11:**
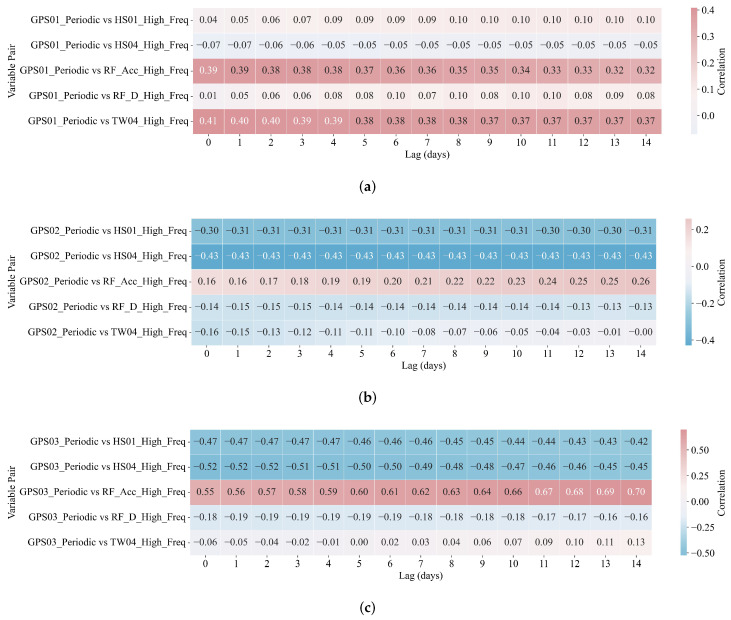
Lagged correlation heatmaps (lags: 0–14 days) for periodic components of ground displacement and environmental variables. All lags are measured in days. (**a**) Periodic: GPS01 vs. environmental covariates. (**b**) Periodic: GPS02 vs. environmental covariates. (**c**) Periodic: GPS03 vs. environmental covariates.

A lagged correlation analysis was employed to quantify the temporal dependencies between ground displacement and its associated environmental drivers. This analysis was performed following VIF-based multicollinearity screening, ensuring that only statistically independent predictors were included (Figure 10 and Figure 11).

The analyses were conducted separately for the trend and periodic subspaces to capture long-term accumulation and short-term variability, respectively. In the trend domain (Figure 10), the correlation heatmaps reveal strong and temporally persistent associations across all three GPS stations (GPS01–GPS03). The displacement trends showed consistently strong negative correlations with RF_D_Low_Freq and HS01_Low_Freq, often exceeding −0.98 in magnitude. This indicates that reduced rainfall and soil moisture are closely associated with gradual subsidence. Conversely, positive correlations emerged with TW03_Low_Freq and TW04_Low_Freq, particularly at GPS02. At that station, the correlation with TW03_Low_Freq peaked at r=0.933 at lag 0, implying that elevated soil temperatures may directly contribute to downward displacement.

For the periodic component (Figure 11), GNSS displacements showed meaningful short-term associations with cyclic environmental signals. Notably, GPS03 exhibited a peak correlation of r=0.656 with RF_Acc_High_Freq at a 10-day lag, indicating a delayed displacement response to accumulated rainfall. Similarly, significant negative correlations with HS04_High_Freq and HS01_High_Freq were detected at GPS02 and GPS03. These patterns are consistent with seasonal oscillations in soil moisture content. Although GPS01 displayed comparatively weaker periodic responses, it still exhibited moderate correlations with RF_Acc_High_Freq and TW04_High_Freq within lag windows of 0–3 days.

### 4.5. Model Prediction Results and Quantitative Performance Evaluation

#### 4.5.1. Trend, Periodic, and Cumulative Component Predictions

**Figure 12:**
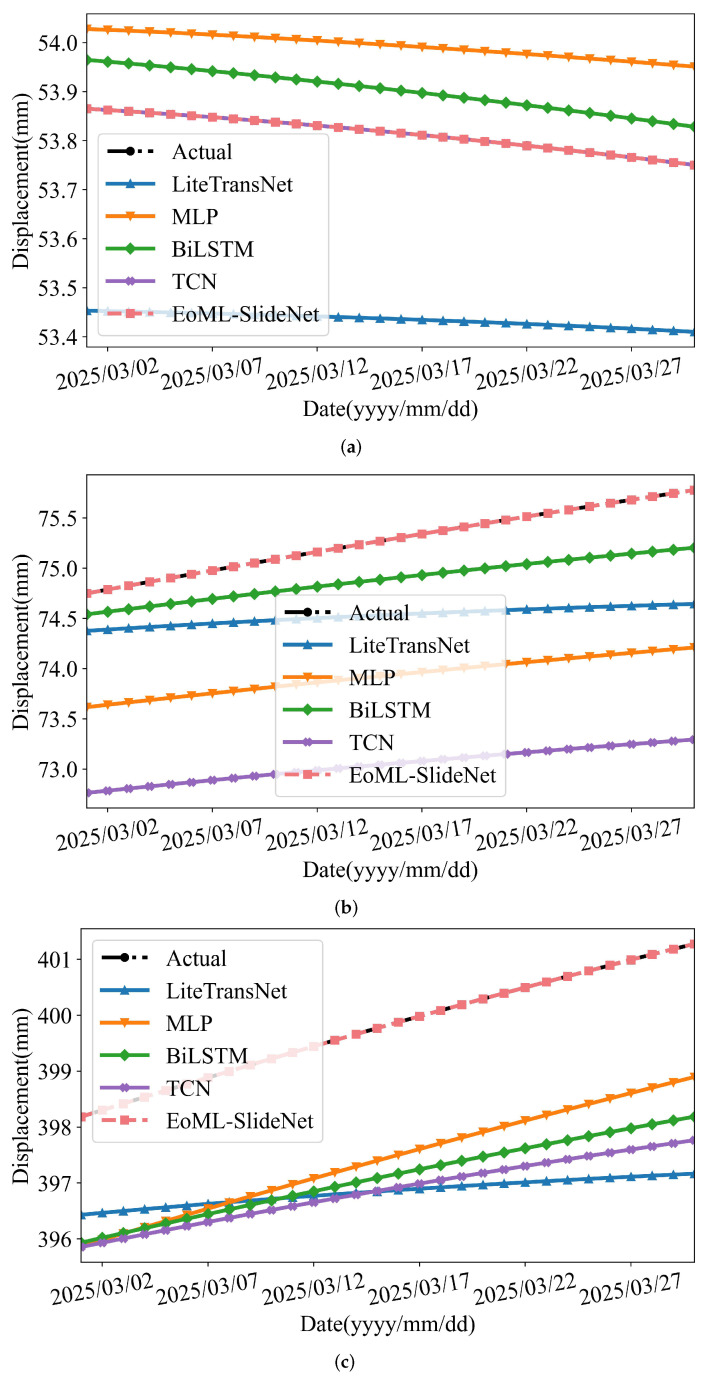
Model predictions versus observations for trend components at GPS01–GPS03. (**a**) GPS01 trend: Model prediction vs. observation for the trend component. (**b**) GPS02 trend: Model prediction vs. observation for the trend component. (**c**) GPS03 trend: Model prediction vs. observation for the trend component.

**Figure 13:**
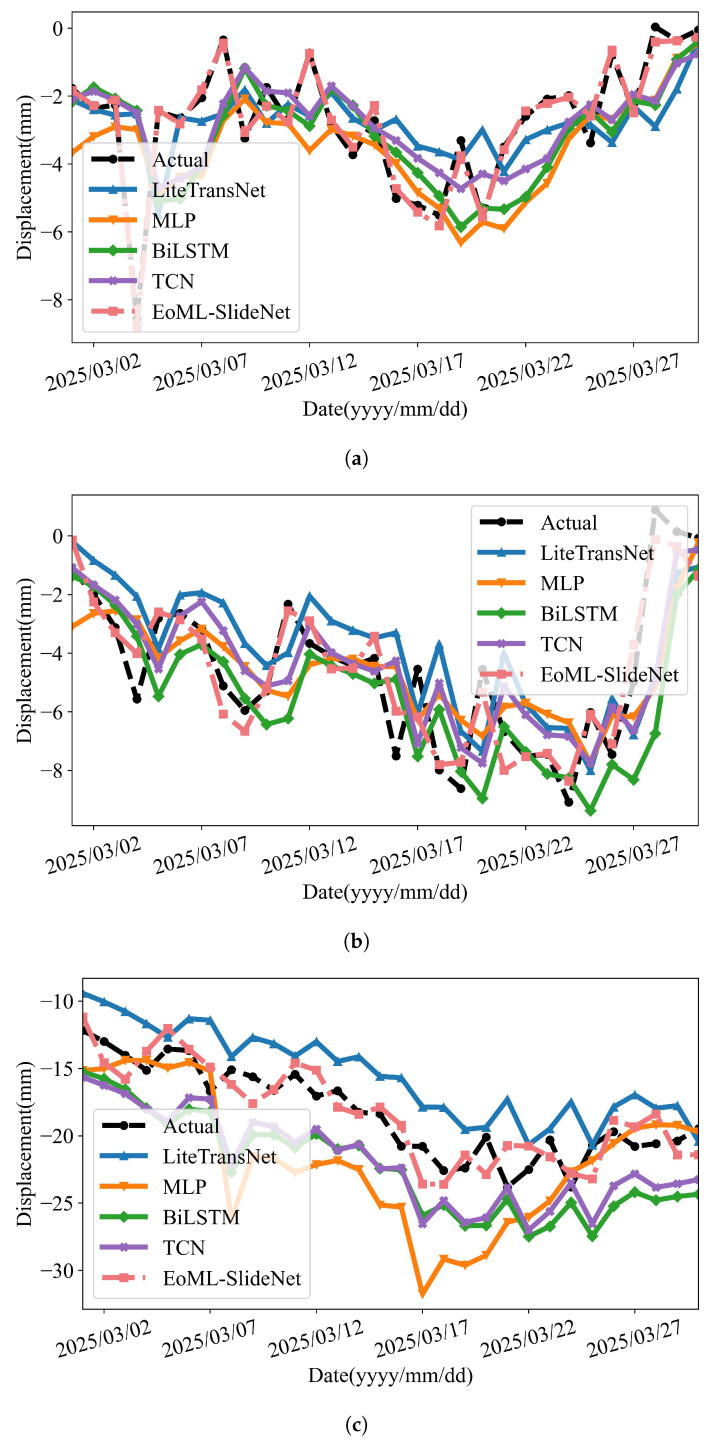
Model predictions versus observations for periodic components at GPS01–GPS03. (**a**) GPS01 periodic: Model prediction vs. observation for the periodic component. (**b**) GPS02 periodic: Model prediction vs. observation for the periodic component. (**c**) GPS03 periodic: Model prediction vs. observation for the periodic component.

Figure 12 and Figure 13 show the predicted and observed displacements. The results include five models: LiteTransNet, MLP, BiLSTM, TCN, and EoML-SlideNet. Each model is evaluated at GPS01, GPS02, and GPS03. The comparisons include three key displacement components: trend, periodic, and cumulative.

As shown in Figure 12, EoML-SlideNet provides the most stable trend predictions, especially near zones of directional change. TCN and MLP generally follow the overall trend but tend to lag or overshoot around inflection points. The LiteTransNet model shows limited adaptability in capturing local transitions, often producing discontinuous fits. In the periodic domain (Figure 13), all deep learning models capture short-term fluctuations to varying degrees. BiLSTM is highly sensitive to localized variations, as seen at GPS03, but this often reduces overall stability. The LiteTransNet model tends to over-smooth periodic peaks due to its global attention mechanism. In contrast, EoML-SlideNet balances local responsiveness and noise suppression, preserving key oscillatory features with high fidelity.

#### 4.5.2. Quantitative Evaluation

**Figure 14:**
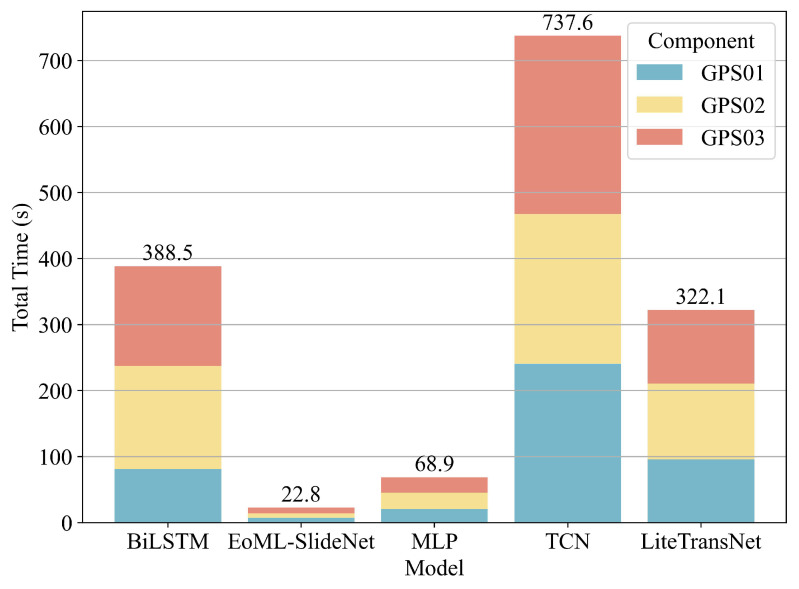
Total inference time (in seconds) for each model aggregated over GPS01, GPS02, and GPS03.

Each model is evaluated in terms of predictive accuracy and computational efficiency, as summarized in Table 6 and Table 7. Figure 14 shows the total inference latency for GPS01, GPS02, and GPS03, providing a cumulative view of runtime overhead.

In terms of predictive accuracy, EoML-SlideNet consistently outperformed all baseline models. For cumulative displacement forecasting (Table 7), it achieved an average $R^{2}$ of 0.9949 across all stations, surpassing the best neural baseline, TCN ($R^{2}=0.9497$). It also yielded the lowest MAE and RMSE in most displacement components. For instance, GPS01_Cumulative recorded an MAE of 0.3502 and RMSE of 0.4331, while GPS02_Cumulative showed an MAE of 0.5567 and RMSE of 0.7190.

From a computational standpoint, deep learning models incurred substantial runtime due to sequential depth and heavy tensor operations. TCN had the longest cumulative inference time (737.6 s), followed by BiLSTM (388.5 s) and LiteTransNet (322.1 s). Even the simpler MLP required 68.9 s, revealing scalability limitations in multi-component scenarios. In contrast, EoML-SlideNet completed all six sub-tasks for the three GPS stations in just 22.8 s. This was 17 times faster than TCN, 14 times faster than LiteTransNet, and nearly 3 times faster than MLP.

**Figure 15:**
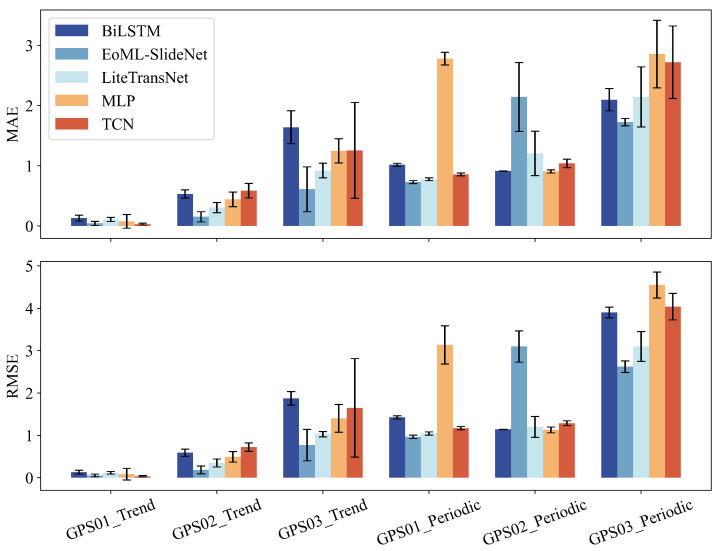
Model generalization and error variance across components and GPS stations under blocked time-based validation.

To assess overfitting and generalization, we conducted robustness testing using a sliding-window validation scheme. For each GPS station, the time series was divided into multiple non-overlapping segments. In each segment, 80% of the data was used for training and 20% for testing. Models were retrained independently on each window to simulate forward prediction under varying real-world conditions. Figure 15 shows the MAE and RMSE distributions across all runs, separately for the trend and periodic components. Error bars represent the standard deviation across validation splits. Compared to the baselines, EoML-SlideNet consistently produced lower mean errors and smaller variance. In contrast, BiLSTM and MLP showed larger error spreads, especially in the periodic component. LiteTransNet tended to over-smooth local variations due to its global attention design, while TCN exhibited instability at GPS03, as reflected by wider error bars.

## 5. Discussion

The proposed EoML–SlideNet framework combines FB–EWT decomposition with lightweight statistical and neural components. It integrates RRX-based trend modeling and the LiFFT block for periodic dynamics. Sudden dynamic changes, such as rainfall-driven acceleration or slope instability, are addressed through two mechanisms. FB–EWT decomposition isolates high-frequency variations that reflect abrupt displacements, while the LiFFT_Periodic branch incorporates harmonics and exogenous bursts (e.g., rainfall spikes) to enable rapid response without destabilizing trend forecasts. This dual-module design enhances responsiveness under rapidly changing field conditions.

The framework achieves high predictive accuracy with a computational cost below 0.016 MFLOPs. Unlike prior models based on computationally intensive networks like LiteTransNet or multi-layer MLPs, EoML–SlideNet shows that a modular, interpretable pipeline can effectively capture the multi-scale nature of landslide displacement. As shown in Table 6 and Table 7, the framework consistently outperforms these baselines in both accuracy and inference efficiency. Visual comparisons in Figure 12 and Figure 13 further confirm its robustness across varying deformation regimes.

A unified evaluation of computational complexity, model compactness, and runtime confirms the efficiency of EoML-SlideNet. As shown in Table 4, it has the lowest theoretical computational cost among all baseline models. The required FLOPs per forward pass remain below 0.016 MFLOPs across various sequence lengths (*K*) and hidden dimensions (*H*). In comparison, BiLSTM scales quadratically with hidden width due to its $O(KH^{2})$ structure. LiteTransNet incurs even higher cost from its $O(K^{2})$ attention operations. The compact architecture of EoML-SlideNet also reduces memory and storage requirements, making it highly suitable for deployment on edge devices.

These theoretical gains yield clear runtime benefits. As shown in Table 6, EoML-SlideNet consistently achieves the shortest inference times across all GPS stations and displacement components, typically completing each task in under 5 s on a CPU-only system. This is 3 to 30 times faster than other baselines, including TCN, BiLSTM, and LiteTransNet. Importantly, this speed does not reduce accuracy. EoML–SlideNet records the lowest MAE and RMSE and the highest $R^{2}$ values across all settings.

As detailed in Section 2, GPS03 shows the highest displacement variance due to rainfall-induced, step-like movements. These abrupt changes increase prediction errors for all baseline models. By applying FB-EWT to separate frequency bands and using high-frequency rainfall features to drive the LiFFT_Periodic branch, EoML-SlideNet effectively addresses this challenge. The model reduces the MAE of GPS03_Cumulative to 1.90 mm, compared to 4.59 mm for the MLP baseline (Table 7). At the same time, it maintains inference times under 10 s. These results demonstrate the framework’s robustness in handling highly non-stationary, episodic displacement patterns. EoML–SlideNet delivers state-of-the-art accuracy and computational efficiency. However, its robustness under temporal variation and spatial generalization requires further evaluation. Sliding-window validation (Figure 15) reveals low error variance across different time splits, suggesting reduced risk of overfitting. Compared to baselines such as BiLSTM and MLP, EoML–SlideNet remains more stable when training and testing boundaries shift, particularly in high-frequency predictions. This improved stability is attributed to its modular design and the targeted feature selection performed by the DBLE–LV module, which effectively reduces multicollinearity and mitigates the effects of data distribution changes.

EoML–SlideNet is designed for rainfall-induced shallow landslides but can be adapted to other failure regimes due to its modular architecture. For deep-seated landslides, the FB–EWT window can be extended to capture longer-term trends. The RRX_Trend branch may be enhanced by adding a viscous Burgers rheology prior to the ExpSmooth–AR branch, with the viscosity and modulus parameters calibrated from piezometer and extensometer data. The LiFFT_Periodic module can be replaced with a simplified TinyStats cell to model low-frequency periodicity. In the case of earthquake-triggered landslides, an additional impulse-response filter can be introduced. This filter, driven by peak ground acceleration, models coseismic offsets while the trend module remains active. These extensions will be tested in future deployments to evaluate their effectiveness under diverse landslide conditions.

The features selected by DBLE–LV (Figure 9, Figure 10 and Figure 11) are parsimonious and geomechanically meaningful. At low frequency, HS01_Low_Freq (near-surface water content) shows strong negative correlation with displacement trends, consistent with unsaturated soil softening driven by progressive infiltration [65]. RF_Acc_Low_Freq (cumulative rainfall) is positively correlated with displacement, reflecting delayed pore-pressure buildup and basal slip activation in humid karst settings [66]. TW04_Low_Freq (deep-layer temperature) contributes moderately, suggesting thermal control on viscosity and drainage. In the high-frequency band (Figure 11), RF_Acc_High_Freq and HS01_High_Freq explain rapid displacement fluctuations following rainfall bursts. These impulsive responses, especially evident at GPS03, reflect shallow saturation surges and transient shear along weak interfaces (Figure 3). Across all stations, the selected predictors exhibit low multicollinearity (VIF < 5; Figure 10b) and align with known hydro-mechanical processes, supporting both model compactness and field interpretability.

Current validation is based on a four-year dataset that includes three displacement regimes: mild creep at GPS01, slow creep at GPS02, and episodic steps at GPS03. However, GPS01 and GPS02 exhibit relatively small cumulative displacements, with totals of less than 60 mm and 100 mm, respectively. As a result, the training set is dominated by low-amplitude micro-creep and contains few large-magnitude events. This variability is sufficient for evaluating the separation of trend and periodic components, but it limits the model’s ability to generalize to slopes that undergo meter-scale movement. To ensure reliable and low-latency performance in real-time applications, broader validation is needed. This includes testing across different lithologies, failure depths, and triggering mechanisms, as well as incorporating streaming-aware adaptations. While the sliding-window evaluation simulates real-time conditions, long-horizon forecasts may expose limitations under low-signal or extreme weather scenarios, where certain predictors become unavailable. Incorporating uncertainty quantification and adaptive dropout may improve model reliability in these situations. Future work will explore real-time sample-level adaptation, cloud-based fleet fine-tuning, uncertainty-aware triggering, Monte Carlo dropout for uncertainty quantification, a full edge–cloud deployment architecture, and the model’s applicability to displacement inputs derived from UAV/SfM photogrammetry, ground-based InSAR, or low-cost tilt sensors.

## 6. Conclusions

This study presents EoML-SlideNet, a lightweight and interpretable framework for forecasting rainfall-induced landslide displacement. It integrates FB–EWT for signal decomposition, the DALE–LV module for frequency-aware feature selection, and two compact forecasting blocks: RRX for trends and LiFFT for periodic dynamics. EoML-SlideNet has fewer than 7050 trainable parameters and a total inference cost below 0.016 MFLOPs, enabling real-time deployment on low-power CPU platforms such as ARM-based edge devices without GPU acceleration.

The proposed method outperforms deep learning baselines such as LiteTransNet and the three-layer MLP, achieving 2–4× lower MAE and RMSE, and reducing inference time by up to 30× on resource-constrained devices. However, its real-time performance has been validated only under specific conditions. The framework’s modular design ensures transparent feature selection and preserves a clear physical link between predictors and displacement behavior. These characteristics make it suitable for real-world early-warning applications. Nevertheless, further validation is required to confirm its reliability under more diverse and extreme field conditions.

Future work will focus on further testing to ensure the robustness of EoML-SlideNet under both temporal variability and spatial generalization. Although the sliding-window evaluation simulates real-time deployment, long-horizon forecasts may reveal limitations, especially under low-signal or extreme weather conditions. To address these challenges, we plan to incorporate uncertainty quantification and adaptive dropout to improve reliability. Cross-site validation in diverse geological settings, including deep landslides and earthquake-triggered failures, will be essential for demonstrating the model’s versatility. In addition, testing on lower-specification edge devices will help evaluate scalability and adaptability in real-world applications. In practice, this will be implemented using a sliding DFT update combined with overlap-save filtering, which reduces per-sample complexity to O(logL) and limits memory usage to a small ring buffer. These optimizations will help ensure EoML-SlideNet remains reliable for landslide monitoring in diverse geological settings while preserving its low computational cost for edge deployment.

## Figures and Tables

**Table 1:** Architectural configurations of baseline models.

Model	Structure Summary	Activation
TCN	Three TemporalBlocks; each block has two Conv1D layers (kernel =16; dilation =1,2,4); channel width =H; first block downsamples if needed.	ReLU
MLP	fc1: Linear(42→H); fc2: H→H/2; fc3: H/2→1	ReLU
BiLSTM	Two-layer BiLSTM: (6→H×2), then (2H→H×2); fc1: 2H→H/2; fc2: H/2→1.	ReLU,Dropout ^1^
LiteTransNet	2 encoder + 2 decoder layers; each encoder layer has 1 × 4-head attention; each decoder layer has 2 × 4-head attentions; FFN (H→4H→H); output fc: (H→1).	ReLU ^2^

The table lists the core architectural elements of each model. ^1^ Dropout is applied between fc1 and fc2 in the BiLSTM. ^2^ ReLU is used in the feed-forward network of each encoder layer.

**Table 2:** Experimental environment summary.

Component	Specification
Operating System	Windows 10 Pro (Build 26100)
CPU (host)	Intel Core i7-9700, 8 cores, 3.0 GHz
System Memory	32 GB DDR4
Python Environment	Python 3.11.7 (Anaconda)
Deep Learning Framework	PyTorch 2.2.1 (CUDA disabled)
Core Libraries	NumPy, Pandas, Matplotlib, scikit-learn, statsmodels
Inference Protocol	Single-threaded, 128-sample window, 300 runs averaged

**Table 3:** Performance comparison of signal decomposition methods.

Method	RMSE (mm)	ERR (%)	SEAR (%)
ICEEMDAN	0.47	97.4	94.2
CEEMDAN	0.49	97.1	89.3
FB–EWT	0.50	96.9	88.1
EMD	0.53	94.2	78.3
VMD	0.62	93.0	74.5

RMSE: root mean square error; ERR: event recognition rate; SEAR: step event accuracy rate.

**Table 4:** Estimated FLOPs (in MFLOPs) for each model under different sequence lengths (*K*) and model widths (*H*).

K	H	TCN [59]	MLP [60]	BiLSTM [61]	LiteTransNet [62]	EoML–SlideNet
K = 32	32	0.10	0.0264	0.26	7.4	0.0047
	64	0.20	0.0826	1.05	23.0	0.0050
	128	0.39	0.2840	4.19	85.5	0.0055
K = 64	32	0.20	0.0556	0.52	29.7	0.0073
	64	0.39	0.1737	2.10	91.9	0.0075
	128	0.79	0.5973	8.39	341.9	0.0081
K = 128	32	0.39	0.1140	1.05	119.4	0.0124
	64	0.79	0.3560	4.19	367.7	0.0126
	128	1.57	1.2240	16.78	1367.6	0.0132
FLOP Expression		6KHk	$O(H^{2})$	$4KH^{2}$	$4N_{\mathrm{blk}}K^{2}+2KH^{2}$	$O\left(L\log L\right)+O\left(HD\right)+O\left(d_{z}h+hH\right)$

**Table 5:** Station-wise model configuration and performance summary (excluding feature count).

Station	$\lambda_{\mathrm{trend}}$	$\lambda_{\mathrm{periodic}}$	MAE (mm)
GPS01	0.006	0.004	1.03
GPS02	0.005	0.003	0.99
GPS03	0.005	0.003	1.02

**Table 6:** Grouped model performance and computational cost by component.

Component	Model	MAE	RMSE	R^2^	Total Time (s)
GPS01_Trend	TCN	0.1324	0.1435	0.9814	88.3400
	MLP	0.1408	0.1589	0.9771	11.1597
	LiteTransNet	0.1352	0.1678	0.9725	35.4976
	BiLSTM	0.1814	0.2178	0.9571	38.4217
	EoML-SlideNet	0.0100	0.0600	0.9913	2.4700
GPS01_Periodic	TCN	1.2523	1.6364	0.8124	147.7100
	MLP	1.4454	1.8496	0.7603	9.6392
	LiteTransNet	0.8960	1.1680	0.895	60.5069
	BiLSTM	1.2059	1.5691	0.8275	42.9944
	EoML-SlideNet	0.3502	0.4331	0.9769	5.0484
GPS02_Trend	TCN	0.4600	0.5213	0.9966	74.0500
	MLP	1.4452	1.8160	0.9582	12.6132
	LiteTransNet	1.9575	2.1183	0.9960	52.0820
	BiLSTM	1.3120	1.6320	0.9662	69.3486
	EoML-SlideNet	0.0400	0.0100	0.9971	1.7819
GPS02_Periodic	TCN	1.4332	1.7808	0.9606	153.1500
	MLP	1.2210	1.5773	0.9691	12.1232
	LiteTransNet	1.7515	2.1990	0.9399	62.5044
	BiLSTM	1.8031	2.1682	0.9415	86.5432
	EoML-SlideNet	0.5567	0.7190	0.9816	4.5700
GPS03_Trend	TCN	8.1689	8.2562	0.9476	117.6600
	MLP	5.1996	6.8583	0.9639	12.4472
	LiteTransNet	6.5400	6.7970	0.9700	50.5958
	BiLSTM	6.5400	6.7970	0.9645	86.3473
	EoML-SlideNet	0.0100	0.0300	0.9905	1.7322
GPS03_Periodic	TCN	4.1730	6.7443	0.9811	152.2
	MLP	6.1944	8.4873	0.9700	10.9063
	LiteTransNet	7.7661	9.5060	0.9624	60.9022
	BiLSTM	6.3908	7.8661	0.9743	64.9173
	EoML-SlideNet	1.9000	3.5334	0.9918	4.5700

**Table 7:** Grouped model performance for cumulative displacement.

Component	Model	MAE	RMSE	R^2^	Total Time (s)
GPS01_Cumulative	TCN	1.2725	1.6581	0.8731	240.5400
	MLP	1.4862	1.8834	0.8363	20.8000
	LiteTransNet	1.3178	1.7015	0.8664	96.0045
	BiLSTM	1.2301	1.5888	0.8835	81.4161
	EoML-SlideNet	0.3502	0.4331	0.9913	7.5100
GPS02_Cumulative	TCN	1.2382	1.5411	0.9741	227.2000
	MLP	1.4743	1.9099	0.9602	24.7364
	LiteTransNet	3.2901	3.7105	0.8497	114.5864
	BiLSTM	2.8699	3.4372	0.8710	155.8800
	EoML-SlideNet	0.5567	0.7190	0.9944	6.3519
GPS03_Cumulative	TCN	10.4331	11.8284	0.9751	269.8600
	MLP	4.5861	6.0007	0.9936	23.3535
	LiteTransNet	12.6910	13.8207	0.9660	111.4980
	BiLSTM	8.6555	11.7241	0.9755	151.2500
	EoML-SlideNet	1.8995	3.5334	0.9978	8.9655

## Data Availability

The datasets presented in this article are not readily available because they were provided by government departments and contain sensitive geospatial information. Requests to access the datasets should be directed to the first author.
